# Topical use of tranexamic acid can reduce opioid consumption compared with intravenous use for patients undergoing primary total hip arthroplasty: a prospective randomized controlled trial

**DOI:** 10.1186/s12891-023-06576-7

**Published:** 2023-06-03

**Authors:** Lei Shen, Zhenhuan Jiang, Qiang Wang, Wei Xu

**Affiliations:** 1 Department of Orthopaedics, the Second Affiliated Hospital of Soochow University, 1055 Road Sanxiang, Suzhou, Jiangsu, 215004 China; 2 Department of Orthopaedics, the Yixing People’s Hospital, 75 Road Tongzhenguan, Yixing, Jiangsu, 214200 China

**Keywords:** Opioid, Tranexamic acid, Total hip arthroplasty, Pain, Topical

## Abstract

**Background:**

The problem of opioid addiction after total hip arthroplasty (THA) has been widely concerned. Tranexamic acid (TXA) has been shown to be effective in reducing blood loss for patients undergoing THA, but few studies focus on its alleviation of postoperative local pain symptoms. The purpose of this study was to investigate whether topical TXA could reduce early postoperative hip pain for primary THA patients, thereby reducing the use of opioids, and whether local pain is related to inflammatory response.

**Methods:**

In this prospective randomized controlled study, we randomly divided 161 patients into a topical group (*n* = 79) and an intravenous group (*n* = 82). Hip pain was assessed using the visual analogue scale (VAS) score within three days after surgery and tramadol was used for pain relief when necessary. Inflammatory markers such as high-sensitivity C-reactive protein (CRP), erythrocyte sedimentation rate (ESR), interleukin-6 (IL-6), total blood loss and hemoglobin drop were assessed by hematologic tests. The primary outcomes included the VAS score and dose of tramadol from the first to the third day after surgery. The secondary outcomes included the inflammatory markers level, total blood loss and complications.

**Results:**

The pain score and inflammation markers level on the first day in the topical TXA group were significantly lower than those in the intravenous TXA group (*P* < 0.05). The correlation analysis showed that the VAS score on the first day after surgery was positively correlated with the inflammation markers level (*P* < 0.05). The tramadol dose for topical group was lower than intravenous group on the first and second day after surgery. There were no differences in total blood loss between the two groups (640.60 ± 188.12 ml vs. 634.20 ± 187.85 ml, *P* = 0.06). There was no difference in the incidence of complications.

**Conclusion:**

Topical use of TXA could relieve the local pain symptoms and reduce opioid consumption compared with intravenous use for patients undergoing primary THA by reduce the early postoperative inflammatory response.

**Trial registration:**

The trial was registered at the China Clinical Trial Registry (ChiCTR2100052396) on 10/24/2021.

Total hip arthroplasty (THA) is a highly effective surgery for treating end-stage hip diseases such as osteonecrosis of the femoral head [1]. Pain and blood loss are common complications after surgery [2], adequate perioperative analgesia and less bleeding can enable patients to exercise early after surgery, so as to reduce complications and mortality [3, 4]. Tranexamic acid (TXA), a common antifibrinolytic drug, has been shown to be effective in preventing bleeding complications, and in some cases reducing mortality with minimal adverse effects [5]. A recent retrospective study [6] showed that more severe postoperative pain in the operative area for patients with topical TXA was associated with greater opioid use, the mechanism being that TXA produces pain by inhibiting g-aminobutyric acid and glycine receptors in the spinal dorsal horn. However, Lei et al. questioned this finding [7], and Goldstein [8] found TXA is a safe adjunct to decrease postoperative pain and swelling in arthroscopic surgery. Therefore, we proposed a prospective randomized controlled trial to investigate the effect of TXA on postoperative pain after THA. We hypothesized that topical TXA could relieve early postoperative hip pain and reduce the opioid consumption for patients undergoing primary THA.

## Methods

### Patient selection

Patient inclusion criteria: patients underwent THA in our hospital for end-stage osteoarthritis of the hip from November 2021 to October 2022. Exclusion criteria: (1) history of diseases that cause abnormal inflammatory markers; (2) history of hip fracture; (3) history of renal failure; (4) history of cardiovascular and cerebrovascular embolism in recent 1 year; (5) history of stent implantation in recent 1 year; (6) history of deep vein thrombosis or pulmonary embolism; (7) pregnant or nursing women; (8) poor compliance; (9) refuse to participate. Withdrawal criteria: (1) previous history of opioid dependence; (2) violation of treatment principles and poor compliance; (3) failure to cooperate with doctors during medication treatment and loss of follow-up; (4) patients and their families called for the trial to be stopped.

### Sample size

PASS 15.0 (NCSS, Inc., USA) was used to calculate the sample size required for the study. Considering that there are few studies on the correlation between TXA and postoperative pain in the previous literature, the results of the pilot experiment were used for calculation. The results of the preliminary experiment showed that the average pain scores of the topical group were 3.41, 2.83 and 2.56 at 3 days after surgery, the mean pain score of the intravenous group was 3.79, 2.88 and 2.76 at 3 days after surgery. Using a two-tailed test with α = 0.05 and β = 0.2, a total of 140 patients were recruited. To allow for a potential drop-out of 15%, we enrolled 161 patients.

### Randomization method

With the patients’ informed consent, the number of ‘0’ or ‘1’ was randomly generated by a computer, with the number ‘0’ representing intravenous TXA and the number ‘1’ representing topical TXA, after which the subjects were randomized by the research assistant into topical and intravenous TXA groups via sealed, opaque envelopes. Because this study is equipped with drug use supervision personnel to randomize and supervise drug use methods, patients, surgeons, and the rest of the clinicians involved in the treatment were blinded to the methods of TXA use. All clinical information involving TXA was collected and analyzed by independent researchers and statisticians who were not involved in the clinical treatment.

### Drug administration for both groups of patients

For patients in the topical TXA group: 100 ml normal saline was dripped intravenously 10 min before skin incision, while 2 g of TXA mixed in 50 ml normal saline was was injected in the drainage tube after closing the incision, keeping it closed for 3 h, while 3 h after the end of surgery 100 ml normal saline was given by intravenous dripping. For patients in the intravenous TXA group, 1 g of TXA mixed in 100 ml of normal saline was administered intravenously 10 min before the incision, 50 ml of normal saline was injected in the drainage tube after closing the incision, keeping it closed for 3 h, while 1 g of TXA mixed in 100 ml of normal saline was administered intravenously 3 h after the end of the surgery.

### Surgical technique and relevant perioperative treatment

Due to the short duration of primary THA, patients were treated with non-catheter combined spinal-epidural anesthesia or general anesthesia, intravenous prophylactic antibiotics 30 min before surgery, and pre-emptive analgesia with an oral dose of 200 mg celecoxib. The surgery was performed by two orthopedic surgeons with extensive experience in joint replacement surgery, using a posterolateral approach and a non-cemented prosthesis. Anticoagulation with 4000 IU low-molecular weight heparin was started 8 h after the procedure. Antibiotics were administered prophylactically until 24 h after surgery. Postoperative analgesia was treated with parecoxib 40 mg M Bid. Immediately after recovery of lower limb sensory activity, the patients were instructed to perform ankle joint active flexion and extension activities and quadriceps femoris isometric contraction exercises. On the first day after operation, the X-ray film was taken to confirm that there was no periprosthetic fracture, and the patient began to ambulate, and guided the hip joint flexion and abduction functional exercise.

All patients enrolled in the study was assessed for pain level acccording to the Standard Operation Procedure (SOP). The pain score (from 0–10, 0 represents no pain and 10 represents the maximum pain level) was assessed by the Visual Analog Scale (VAS) every 4 h from the recovery of lower limb sensory activity on the day of surgery to the third day after surgery, and the VAS score was compared by daily averages. Oral tramadol sustained-release tablets (50 mg) or multiple doses were used for pain relief if the patient's pain symptoms were severe.

All patients had routine blood tests, coagulation-related indicators and inflammatory indicators including high-sensitivity CRP, IL-6 and ESR reviewed daily for three days after surgery, and imaging data examination and deep vein ultrasound of the lower extremities were performed on the second postoperative day after drainage was removed to rule out deep vein thrombosis. Patients with hemoglobin values less than 70 g/L or between 70 and 100 g/L but with significant symptoms of anemia were treated with red blood cell transfusion to correct anemia. All patients were encouraged to walk immediately on the first postoperative day to reduce adverse complications such as pneumonia and thrombosis caused by bed rest.

### Preoperative data collection, and postoperative outcome evaluation

Demographic data of patients included age, sex, body mass index (BMI), cardiac ASA classification, and concomitant diseases. Preoperative laboratory findings included hemoglobin value (Hb), hematocrit value (Hct), platelet count (PLT), international normalized ratio (INR), and prothrombin time (PT).

The primary postoperative evaluation included the mean VAS score in the surgical area and the tramadol consumption from the day after surgery to the third day after surgery.

The second postoperative evaluation included the level of inflammatory indicators such as CRP, IL-6 and ESR, calculation of hemoglobin drop, total blood loss and postoperative blood transfusion rate for the first day of postoperative routine blood results. The hemoglobin drop was calculated by referring to the daily postoperative routine blood test results, and the total blood loss was calculated by referring to the calculation methods of Gross et al. [9]. Complications such as postoperative deep venous thrombosis, pulmonary embolism and deep infection were all recorded for 3 months after surgery.

### Statistical analysis

Data analysis mainly includes measurement data and enumeration data. The measurement data included age, height, weight, BMI, preoperative hematology-related values, reduction of Hemoglobin, total blood loss and postoperative inflammatory indicators level. The enumeration data included the sex, ASA classification, the opioid consumption transfusion and complications. Shapiro–wilk test was used to test the normality of measurement data, and x ± s was used to show the normal distribution, and independent sample t-test was used for comparison between two groups. Chi‑square test or Fisher exact test were used for enumeration data. Pearson correlation analysis was used to analyze the correlation. All statistical analyses were performed using SPSS 24.0 (SPSS Inc., Chicago, IL, USA), and a value of *P* < 0.05 was considered statistically significant.

## Results

**Fig. 1:**
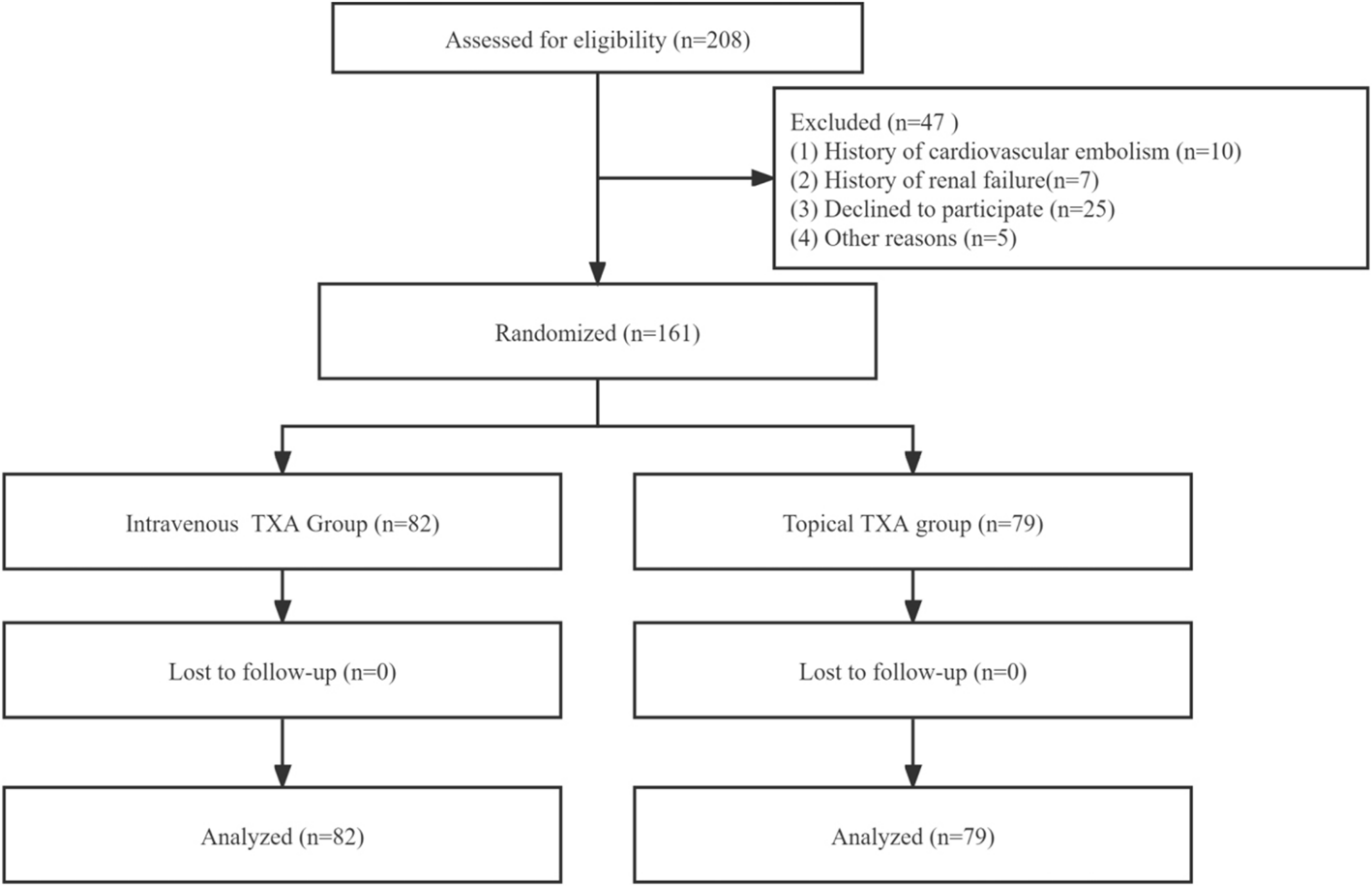
Flow diagram showing participant screening and allocation

A total of 208 patients underwent THA during the study period, and 47 patients were excluded according to the inclusion and exclusion criteria, including 42 patients who met the exclusion criteria, and 5 patients who were excluded for other reasons. The 161 patients were randomly assigned, 79 to the topical TXA group and 82 to the intravenous TXA group (Fig. 1).

There was no statistically significant difference in demographic data such as sex, age, height, weight, BMI, and cardiac ASA grade between the two groups (*P* > 0.05, Table 1). There was also no significant difference in preoperative hematology-related examinations, including Hb level, Hct, PLT count, PT, and INR (*P* > 0.05, Table 1).

**Table 1:** The comparison of demographic data of two groups

Variables	Topical Group (*N* = 79)	Intravenous Group (*N* = 82)	t/x^2^	*P* Value
Age(y)	67.09 ± 4.69	67.63 ± 5.10	0.705	0.482
Sex				
Female	37	42	0.309	0.578
Male	42	40		
Weight(kg)	62.71 ± 6.87	62.54 ± 8.54	-0.141	0.888
Height(m)	1.66 ± 0.06	1.67 ± 0.06	1.918	0.057
BMI(kg/m^2^)	22.16 ± 2.22	21.81 ± 2.45	-0.948	0.344
ASA classification				
1	8	5	1.789	0.617
2	25	33		
3	42	40		
4	4	4		
Preoperative values				
Hemoglobin(g/L)	124.48 ± 12.81	122.67 ± 13.83	-0.861	0.391
Platelet count((× 1000/mm^3^)	230.86 ± 62.28	245.62 ± 64.37	1.478	0.141
Hematocrit(%)	41.80 ± 3.92	40.53 ± 4.92	-1.806	0.073
INR	1.06 ± 0.11	1.06 ± 0.12	0.329	0.742
Prothrombin time(sec)	10.95 ± 0.93	10.88 ± 0.86	-0.498	0.619

**Fig. 2:**
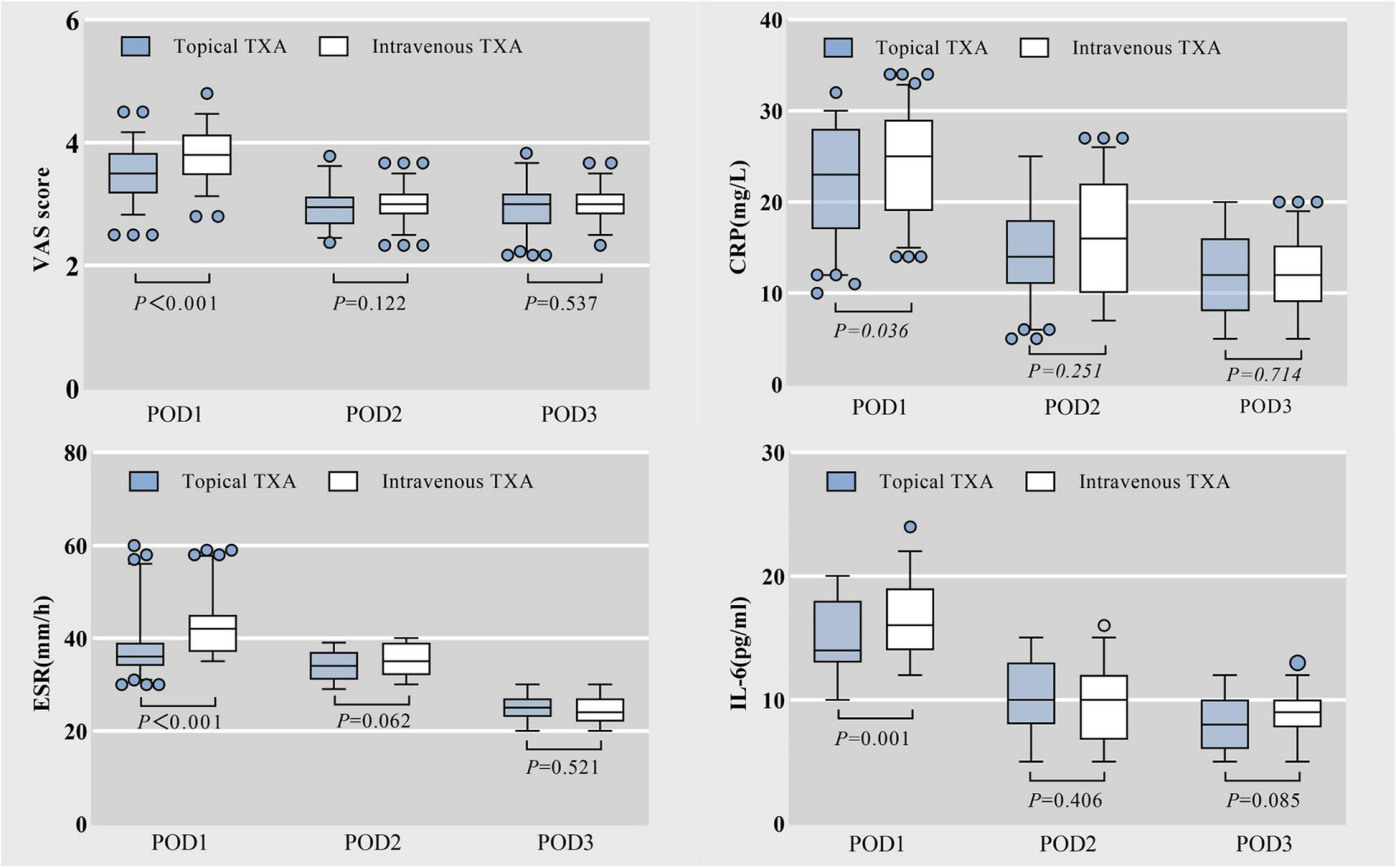
The comparation of VAS socre and inflammatory indicators level (CRP, IL-6, ESR) between two groups

**Fig. 3:**
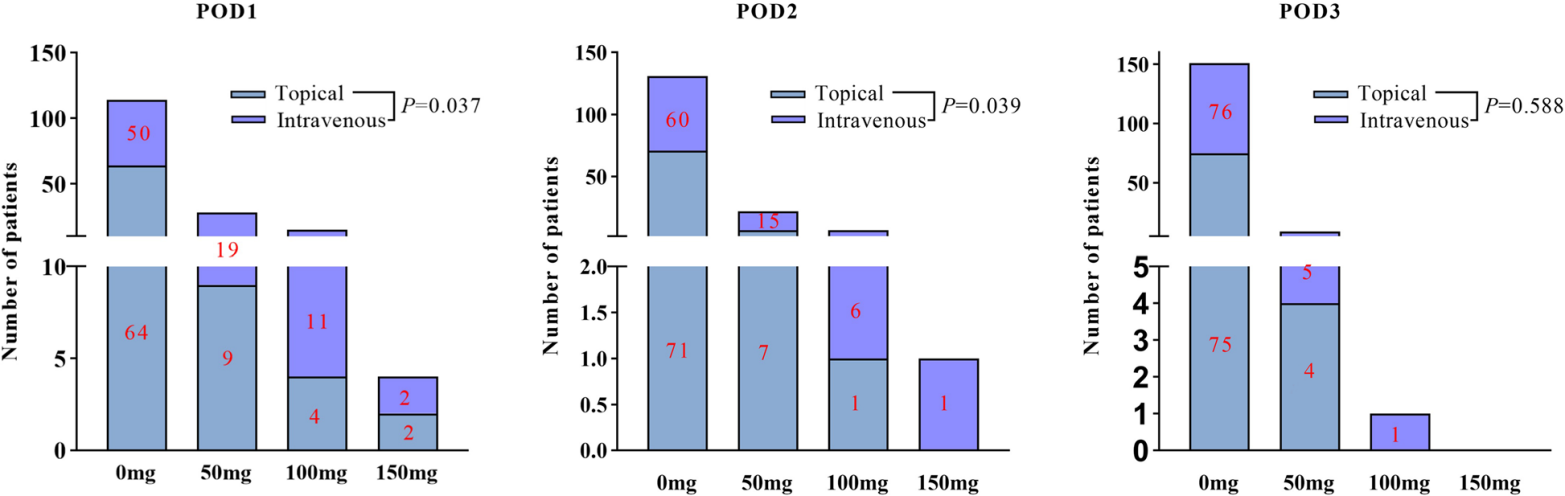
The comparation of tramadol consumption between two groups

The hip pain score on the first day after surgery in the topical TXA group was significantly lower than that in the intravenous TXA group (3.54 ± 0.46 vs. 3.84 ± 0.39, *P* < 0.001, Fig. 2). The tramadol consumption of the topical group was lower compared with intravenous group on the first and second day after surgery (*P* = 0.037*, P* = 0.039, Fig. 3).

**Fig. 4:**
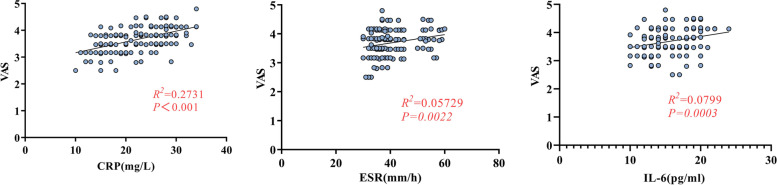
The scatter diagram of the CRP, ESR, IL-6 with VAS score 1 day after surgery, correlation analysis was used to analyze the associations

The CRP, IL-6 and ESR level on the first day after surgery in the topical TXA group were significantly lower than that in the intravenous group (*P* = 0.036*, P* < 0.001, *P* = 0.001, Fig. 2). The correlation analysis showed that the VAS score on the first day after surgery was positively correlated with the CRP, IL-6 and ESR level (*P* < 0.05, Fig. 4) and the scatter diagram of the three inflammatory indicattors with VAS score in all patients were showed in Fig. 4. There was no significant difference in VAS score or inflammatory indicators level between the two groups on the second and third days after surgery (*P* > 0.05, Fig. 2).

The postoperative evaluation showed no significant difference in the decrease of Hb values between the two groups (12.22 ± 4.05 g/L vs. 17.07 ± 4.04 g/L, *P* = 0.824). There was also no significant difference in the total blood loss calculated between the two groups (640.60 ± 188.12 ml vs. 634.20 ± 187.85 ml, *P* = 0.827). Seven patients in the topical group and ten patients in the intravenous TXA group received blood transfusions postoperatively, with no statistically significant difference (*P* = 0.491).

In the topical group, 1 patient developed intramuscular venous thrombosis after surgery, and was given hypodermic anticoagulant therapy with 4000 IU Q12h low-molecular weight heparin after a specialist consultation. One week later, re-examination showed that the thrombosis had disappeared. There were no postoperative complications such as pulmonary embolism or incision infection in the 2 groups (*P* > 0.05).

## Discussion

Currently, there remains controversy regarding the perioperative use of TXA in THA, and few studies investigated the effect of TXA on postoperative hip pain in patients undergoing THA. Opioid abuse is currently a medical concern due to pain after total hip replacement [10]. In this study, we found that the topical use of TXA could reduce the inflammatory response in the early postoperative period, as shown by the lower CRP, IL-6 and ESR level in the early postoperative period, and the VAS score of the hip as well as the tramadol consumption were also significantly lower in the topical TXA group compared to the intravenous TXA group.

In order to promote the rapid recovery process of patients after surgery, pain management is an important part that cannot be ignored. THA is one of the most common joint replacement surgeries in orthopedics, and numerous studies have been focused on reducing the amount of opioids used during the perioperative period [11, 12]. Reports on the use of opioids after orthopedic surgery vary greatly, and only a few guidelines have standardized the duration and dose of opioid use [13, 14]. Postoperative opioid abuse may lead to various complications after primary THA, such as increased mortality, revision surgery and thromboembolic events [15–17]. A cohort study conducted by Namba [18] showed use of medium–low (100–219 mg) amounts of oral morphine equivalents in days 91–180 after surgery was associated with a 6 times higher adjusted risk of 1 year revision. Essex et al. [19] found that reduction in morphine consumption could reduce the incidence of postoperative hypoxia and asphyxia. Due to the growing problem of opioid abuse, Prentice et al. [20] conducted a cohort study to investigate the preoperative risk factors leading to opioid use after total hip arthroplasty, the result showed that the risk factors led to greater utilization in days 91 to 360 including higher body mass index, female sex, acquired immunodeficiency syndrome and peripheral vascular disease. Wagner et al. [21] found that the use of liposomal bupivacaine during THA reduced postoperative opioid use as well as the length of hospital stay. A prospective randomized controlled trial conducted by Lucero [22] showed that additional dose of dexamethasone after surgery could significantly relieve pain and reduce tramadol consumption for patients undergoing primary THA. Therefore, it is necessary to find effective measures to prevent opioid abuse and reduce the incidence of various adverse complications.

Previous studies [23–25] have shown that TXA could optimize pain control for orthopaedic surgery such as arthroscopic surgery, joint replacement surgery and joint arthrolysis surgery, but there is still controversy about whether TXA increases or decreases pain. The study conducted by Jeffery [6] showed that topical use may lead to increased local postoperative pain and opioid use. However, Lei [7] questioned the results of this study and argued that previous studies have shown that topical TXA can reduce pain in the surgical area, while the use of topical TXA was not well described in the study. A retrospective matched case–control study conduceted by Remérand [26] concluded that TXA decreases risk of haematomas but not pain after hip arthroplasty. For the patients receiving TXA, more morphine was consumed on the seventh postoperative day, but authors thought that beneficial effects in terms of blood sparingfar outweigh the small increase in morphine consumption. Based on previous studies, the controversy about whether TXA can reduce pain may be related to the following reasons: (1) for local use, TXA and normal saline are injected into the joint cavity by the drainage tube. Due to the abundant pain receptors around the hip joint are sensitive to pressure changes [27], a large amount of normal saline will lead to increased local pressure of the hip joint, triggering pain receptors and causing pain; (2) the local hemostatic effect was related to the dose of TXA [28, 29], increased local bleeding leads to soft tissue swelling, which causes pain; (3) the use of more normal saline lead to a lower local concentration of TXA, which weakens the hemostatic effect according to the concentration-drug effect ratio, leads to increased local bleeding, increased tissue swelling and pain; (4) excessive less use of normal saline causes the TXA to be not evenly diffused into the whole joint cavity, that weakens the hemostatic effect and leads to more bleeding and pain. 

Since the generation and perception of pain is a complex physiological process, above studies have drawn different conclusions. In clinical treatments, pain is a multifactorial associated symptom. In previous studies [30, 31], TXA could relieve pain symptom by reducing inflammatory response after hip and knee replacement surgery. Wang et al. [30] found that oral administration of 1 g TXA at 3 h, 7 h, 11 h, and 15 h after surgery minimized postoperative bleeding and inflammation in knee joint replacements, thereby reducing pain and promoting rapid recovery. Okholm et al. [32] showed that perioperative use of TXA in orthopedic patients was associated with decreased C-reactive protein and interleukin-6 levels compared to those who did not receive or received lower doses of TXA. Similar to the above findings, in our study, CRP level (22.09 ± 5.68 vs. 24.01 ± 5.84, *P* = 0.036), IL-6 level (14.87 ± 3.14 vs. 16.51 ± 3.09, *P* = 0.001) and ESR level (38.65 ± 7.51 vs. 42.84 ± 6.28, *P* < 0.001) on the first postoperative day were lower in the topical group than in the intravenous group, which has some correlation with the topical use of TXA inhibiting the local surgical area inflammatory response. Because the inflammatory reaction was inhibited and the patient’s local pain and irritation symptoms were improved, the VAS score on the first day after surgery was lower in the topical TXA group than in the intravenous TXA group (3.54 ± 0.46 vs. 3.84 ± 0.39, *P* < 0.001). Due to the improvement of postoperative pain symptoms, tramadol use in the topical group was less than that in the intravenous group on the first and second days after surgery. It is worth noting that this study only suggests the positive effect of early topical use in a short period of time. Patients undergoing THA could immediately be taken out of bed and walk the day after surgery for rehabilitation. The amount of exercise varied in each patient due to many confounding factors, and the pain-reducing effect of TXA could not be judged. If patients still have significant pain, it is necessary to consider abnormalities of the incision, and a high index of inflammation prompts evaluation for possible deep infection.

As the most common perioperative complication of THA, blood loss has received extensive attention. Standardized blood management has great benefits for early postoperative rehabilitation and functional exercise of patients. As an antifibrinolytic drug, TXA has been confirmed to reduce postoperative blood loss and the rate of blood transfusion in many studies over the past decade [33, 34]. For efficacy, a meta-analysis by Fang [35] found that although topical and intravenous use of TXA had equivalent efficacy in reducing postoperative blood loss and reducing the blood transfusion rate, the postoperative hemoglobin loss was lower after intravenous use. However, a prospective study conducted by Zhang [36] found that topical use of TXA was more effective, although both methods reduced bleeding volume. In this study, we found no significant difference in postoperative total blood loss and postoperative transfusion rate between the two methods, but hemoglobin reduction was less in the intravenous group than in the topical group, which was also similar to the findings of Sun [34] and Fang [35].

The strength of this study is that it is a prospective randomized controlled trial to compare the VAS score three days after surgery between the two TXA methods, combined with CRP, IL-6 and ESR levels for a comprehensive assessment. However, this study also has some limitations. First, all patients were treated with oral tramadol sustained-release tablets if the conventional analgesic treatment was not effective, but not all patients were sensitive to this drug; second, patients with coagulation dysfunction, such as taking aspirin, were not included. It has been confirmed that TXA is also safe and effective in patients taking aspirin for a long time [37]. As an antipyretic and analgesic drug, further studies are needed to determine whether preoperative administration of aspirin will affect postoperative opioid use. Finally, postoperative hip pain may also be related to local swelling. We will further evaluate the effect of local manifestations of the hip on pain in the future.

## Conclusion

In this study, we found that topical TXA use (2 g of TXA mixed in 50 ml normal saline injected in the drainage tube after closing the incision, keeping it closed for 3 h) could relieve local pain symptoms and reduce opioid consumption compared with intravenous TXA for patients undergoing primary THA. In addition, the effect of reducing blood loss was equivalent for the two usages. 

## Data Availability

The datasets generated and analysed during the current study are not publicly available due to limitations of ethical approval involving the patient data and anonymity but are available from the corresponding author on reasonable request.
